# Assessment of vitamin B6 status in never-pregnant, pregnant and postpartum women and their infants

**DOI:** 10.1007/s00394-022-03033-4

**Published:** 2022-11-01

**Authors:** Anne-Lise Bjørke-Monsen, Kristin Varsi, Sunniva Todnem Sakkestad, Arve Ulvik, Per Magne Ueland

**Affiliations:** 1 Laboratory of Medical Biochemistry, Innlandet Hospital Trust, Lillehammer, Norway; 2 Department of Medical Biochemistry and Pharmacology, Haukeland University Hospital, Bergen, Norway; 3 Bevital AS, Bergen, Norway

**Keywords:** Vitamin B6, Pregnancy, Postpartum, Lactation, Infants, Women

## Abstract

**Purpose:**

Pyridoxal 5´-phosphate (PLP) is the main form of vitamin B6 in humans and functions a coenzyme for more than 160 different enzymatic reactions. The purpose of the study was to find plasma PLP concentrations, which ensure an optimal vitamin B6 status determined by a metabolic marker, in never-pregnant, pregnant and lactating women and their infants.

**Methods:**

In an observational, prospective study, plasma PLP and the metabolic marker, HKr (hydroxykynurenine/(kynurenic acid + anthranilic acid + xanthurenic acid + hydroxyanthranilic acid) were assessed in women (*n* = 114) from pregnancy week 18 to 6 months postpartum and related to infant status. Never-pregnant women 18–40 years (*n* = 127) were included as controls.

**Results:**

Compared to controls, plasma PLP decreased during pregnancy and increased postpartum, while HKr increased from week 18 to 6 weeks postpartum, indicating maternal vitamin B6 insufficiency during this period. In never-pregnant women, HKr increased gradually with plasma PLP < 100 nmol/L, and in pregnancy week 28 a sharp increase in HKr was seen at plasma PLP < 30 nmol/L. Despite correcting for maternal vitamin B6 status, infant median plasma PLP decreased with months of exclusive breastfeeding.

**Conclusions:**

Plasma PLP and kynurenine concentrations differ substantially between never-pregnant, pregnant and postpartum women and infants. A plasma PLP concentration in the range of 50–100 nmol/L seems to ensure an optimal vitamin B6 status for never-pregnant women, whereas a plasma PLP > 30 nmol/L in pregnancy week 28 ensures an adequate vitamin B6 status during pregnancy and lactation. Infant vitamin B6 status at age 6 months is inversely correlated to number of months of exclusive breastfeeding.

**Supplementary Information:**

The online version contains supplementary material available at 10.1007/s00394-022-03033-4.

## Introduction

All organisms are dependent on vitamin B6, but only microorganisms and plants are able to synthesize it de novo, and humans rely on intake of the vitamin from food. Major sources of vitamin B6 in the diet are fish, meat, offal, potatoes, and milk and dairy products.

Pyridoxal 5´-phosphate (PLP) is the main form of vitamin B6 in animal tissue and makes up 70–90% of the total vitamin B6 in plasma. PLP is a coenzyme for more than 160 different enzymatic reactions in the metabolism of amino acids, carbohydrates, lipids and neurotransmitters [1]. An optimal status is important, particularly during periods of rapid growth and development.

Poor vitamin B6 status appears to decrease the probability of conception and to increase the risk of early pregnancy loss [2]. In animal studies, severe maternal vitamin B6 deficiency has been associated with lower body weight, skeletal defects, convulsions and impaired neuromotor development in the offspring [3, 4], but no such associations have been documented in humans [5].

Plasma PLP is reported to decrease throughout pregnancy, whereas the concentrations in newborns are reported to be significantly higher than in the mothers, suggesting fetal sequestration of the vitamin [6, 7]. Maternal supplementation with vitamin B6 produces a rapid increase in milk concentrations of all the different B6 vitamers [8], and vitamin B6 intake of breastfeeding mothers has been shown to be a strong predictor of infant status [9].

It is currently unknown whether the reported decline in plasma PLP concentrations during pregnancy is due to maternal deficiency or merely reflect a physiological pregnancy-induced response [5].

**Fig. 1:**
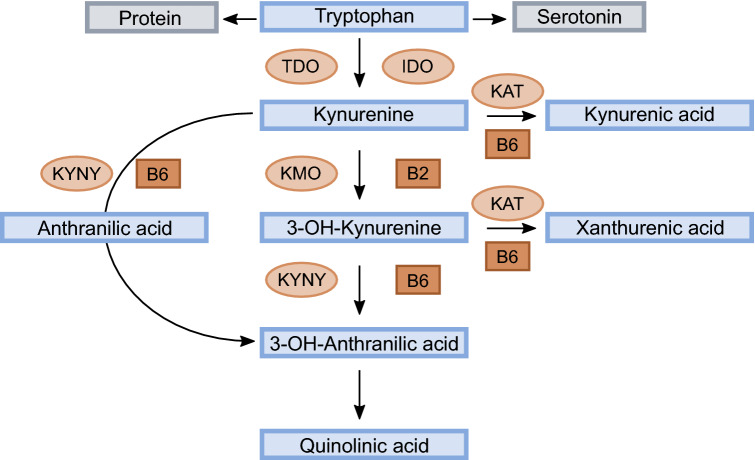
Schematic drawing of the kynurenine metabolism. Pyridoxal 5´-phosphate (PLP) is a cofactor in the conversion of kynurenine (Kyn) to kynurenic acid (KA) by kynurenine aminotransferase (KAT), and to anthranilic acid (AA) by kynureninase (KYNU), as well as in the conversion of 3-hydroxykynurenine (HK) to either xanthurenic acid (XA) by KAT or to 3-hydroxyanthranilic acid (HAA) catalyzed by KYNU

Plasma PLP is the most commonly used marker of B6 status, but is reduced during inflammation and is also affected by renal function and smoking [1]. The HKr index, defined as 3-hydroxykynurenine (HK) and divided by the sum of kynurenic acid (KA) + anthranilic acid (AA) + xanthurenic acid (XA) + hydroxyanthranilic acid (HAA), is a measure of the overall effect of PLP in the kynurenine pathway (Fig. 1) and proposed to be a functional marker of vitamin B6 status [1, 10].

Data on vitamin B6 status during pregnancy and the postpartum period are scarce. In this observational, prospective study, we assessed plasma PLP and the functional marker HKr in healthy never-pregnant, pregnant and postpartum women and their infants with the purpose of identifying plasma PLP concentrations which ensures an optimal vitamin B6 status, as determined by low levels of the functional marker HKr.

## Materials and methods

### Study population and design

This was an observational, prospective study developed to investigate micronutrient status, including vitamin B6, during pregnancy and lactation and the associated infant micronutrient status at 6 months. The population included 140 healthy women with a singleton pregnancy, who were recruited in pregnancy week 18 at routine ultrasound examination at the Obstetrical Department at Haukeland University Hospital, Bergen, Norway. The women were followed week 28 and 36 in pregnancy, and 6 weeks, 4 and 6 months postpartum. The final visit also included the infant. Women with pregnancy-related or chronic disease were excluded, except those with well-regulated hypothyroidism (*n* = 7). Of the 140 pregnant women initially recruited, 114 met the inclusion criteria, and attended all visits in the present study. During the same period, 127 healthy, never-pregnant women between 18 and 40 years old were recruited as controls among students and employees at the University of Bergen and Haukeland University Hospital, Bergen, Norway.

The calculation of the sample size was based on data from our previous studies on cobalamin status in infants [11, 12] with the assumption that a good maternal cobalamin status in pregnancy would result in tHcy concentrations in the lower quartile (< 6.15 μmol/L) for infants at 6 months. A calculated sample size of 65 would give the study a statistical power of 90% to detect a 25% relative difference in infant tHcy levels. On the basis of our experience from earlier studies, a high dropout rate was expected, and as obtaining blood samples from infants may be difficult, a total of 140 pregnant women were recruited.

Ethical approval of the protocol was granted by the Regional Committee on Medical Research Ethics, REK 2011/2447, and written informed consent was obtained from all women.

### Clinical data

At each visit, the participants completed a questionnaire applied by an interviewer (first and second author) concerning age, years of completed education, parity (Para 0 = no previous births), health status, diet, including fish consumption and use of multiple micronutrient supplements (MMN), alcohol and tobacco. At the first visit self-reported height was recorded and the participant were asked about weight at each visit. The postpartum visits included additional information about infant nutrition and growth parameters.

Use of MMN among the pregnant women was recorded at eight time points; before pregnancy, in the first trimester, at pregnancy week 18, 28 and 36, and 6 weeks, 4 and 6 months postpartum. Use of MMN more than 3 days per week at five or more of these eight time points was defined as a regular supplement user, use at one to four time points as an occasional supplement user and no use at any time point as a non-user. For the controls, use of MMN more than 3 days per week was defined as a regular MMN supplement user.

### Blood sampling and analysis

At each visit, non-fasting blood samples were obtained by antecubital venepuncture and collected into vacutainer tubes without additives (Terumo). Blood samples were available for all mothers at all-time points except for week 18 of pregnancy (missing *n* = 7), and for the majority of the infants at age 6 months (104/114, 91%).

Analysis of plasma PLP and kynurenines was performed by liquid chromatography on an Agilent series 1100 HPLC system coupled with electrospray ionization tandem mass spectrometry (ESI–MS/MS) on an API 4000 triple-quadrupole tandem mass spectrometer from Applied Biosystems/MSD SCIEX [13].

### Statistical analysis

A normality test (Shapiro–Wilk test) was used to determine normality of variables. Age is presented as mean and standard deviation (SD) and compared by Student’s *t* test. Concentrations of plasma PLP and kynurenines are presented as median and interquartile range (IQR), and compared by Mann–Whitney *U* test or Kruskal–Wallis test. Chi-squared test was used for categorical data like body mass index (BMI) categorized into four categories (< 18.5, 18.5–24.9, 25–29.9, ≥ 30), educational level, smoking status and use of MMN.

Spearman correlations and multiple linear regression models were used to explore relationships between parameters. Maternal age, parity, use of MMN, as well as infant gender, weight at birth and weight increase to 6 months, factors known to modify serum concentrations of substances that are transferred from mother to child during pregnancy and lactation[8, 11, 14], were included in the multiple linear regression models.

Graphical illustrations of the relationships between PLP and HKr were obtained by generalized additive models (GAM). Visual inspection of the GAM models and possible breakpoints of HKr concentration increases was done to establish possible adequate cut-offs for plasma PLP in the different groups in the study. Generalized additive models of the relation between infant plasma PLP and months of exclusive breastfeeding was adjusted for maternal plasma PLP in pregnancy week 28 and use of MMN throughout pregnancy and lactation.

The SPSS statistical program (version 26) and the packages “mgcv” in R, version 4.0.4. (The R Foundation for Statistical Computing) were used for the statistical analyses. Two-sided *P *values < 0.05 were considered statistically significant.

## Results

### Demographics

Baseline demographic characteristics of the never-pregnant women (*n* = 127) and pregnant women (*n* = 114) are shown in Table 1. Most never-pregnant and pregnant women were healthy with a normal BMI (< 25 kg/m^2^) and had higher education. All mothers and controls had an omnivore diet. Forty-seven percent of the mothers (54/114) were defined as a regular user of MMN supplements, 39% (45/114) as an occasional user and 13% (15/114) as a non-user. The majority of the mothers (99/114) reported use of the same brand of MMN for pregnant women containing pyridoxine 1.4 mg/tablet, and the same brand for lactating mothers containing pyridoxine 1.5 mg/tablet. A lower percentage of the controls were regular MMN users compared to the pregnant women, and the pyridoxine content in the supplements used in this group varied from 1.3 to 2 mg/tablet.

**Table 1:** Baseline characteristics of healthy never-pregnant women and pregnant women in week 18 of pregnancy

	Never-pregnant women	Pregnant women	*P* value
Number	127	114	
Age, y, mean (SD)	25.3 (4.7)	31.5 (4.3)	< 0.001^1^
Prepregnancy BMI^2^, *n* (%)	22.4 (3.0)	22.8 (3.1)	0.32^1^
< 18.5 kg/m^2^	6 (5%)	4 (4%)	0.88^3^
18.5–24.9 kg/m^2^	96 (78%)	85 (76%)	
25–29.9 kg/m^2^	18 (15%)	20 (18%)	
≥ 30 kg/m^2^	3 (2%)	3 (3%)	
Higher education, *n* (%)	88 (69%)	67 (59%)	0.11^3^
Para 0, *n* (%)	127 (100%)	63 (55%)	< 0.001^3^
Smoking, *n* (%)	2 (2%)	2 (2%)	0.92^3^
Regular use of multiple micronutrient supplements (≥ 3 days/week), *n* (%)	27 (21%)	54 (47%)	< 0.001^3^

^1^Comparison by Student’s *t* test

^2^BMI, Body mass index

^3^Comparison by Pearson chi-squared test

The infants were all healthy, born at term (mean gestational age 39.9 (SD 0.8), range 38–42 weeks), with an appropriate for gestational age weight, mean 3573 (SD 418) grams and 53% (60/114) were males. In a multiple linear regression analysis, parity was the only significant predictor for birth weight (B: 125 g, Beta: 0.25, *p* = 0.03), in a model which additionally included maternal age, prepregnancy BMI, use of MMN, plasma PLP and HKr in pregnancy week 28 and infant gender. At age 6 months, the weight had increased by mean 125% (SD 30) to a mean weight of 7969 (SD 987) grams.

Mean duration of exclusive breastfeeding was 3.8 (SD 1.5) months, but the majority (86%) were still breastfed at 6 months. Approximately 60% of the infants had been introduced to solid food at 4 months, and four infants (4%) were still exclusively breastfed at 6 months.

### Plasma PLP and kynurenines in never-pregnant, pregnant and postpartum women

Data for plasma PLP and kynurenines are shown in Table 2. Compared to never-pregnant controls, median plasma PLP concentration was reduced by 39% in pregnancy week 18 and progressively declined further throughout pregnancy (*P* < 0.001). Postpartum, median maternal plasma PLP concentration increased until 4 months, when it was 32% higher than in never-pregnant women (*p* = 0.04). At 6 months postpartum there was no difference between never-pregnant and postpartum women (*p* = 0.10).

**Table 2:** Plasma pyridoxal 5-phosphate and kynurenine concentrations in never-pregnant (*n* = 127), pregnant and postpartum women (*n* = 114) and their infants at 6 months of age (*n* = 104)

Plasma parameters Median (25th, 75th percentile)	Never-pregnant women (*n* = 127)	Pregnant women (*n* = 114)			Postpartum women (*n* = 114)			Infants (*n* = 104)
		Week 18	Week 28	Week 36	6 weeks	4 months	6 months	
Pyridoxal 5-phosphate, nmol/L	64.5 (50.3, 99.2)	40.1 (26.5, 55.9)	29.9 (20.4, 41.7)	23.0 (17.6, 30.6)	65.4 (44.7, 95.1)	86.1 (50.2, 121.5)	78.2 (51.7, 118.5)	143.5 (93.4, 219.0)
HKr (no unit)^1^	33 (28, 38)	37 (32, 44)	42 (33, 51)	46 (37, 62)	47 (38, 59)	37 (32, 42)	34 (30, 40)	85 (68, 108)
3-Hydroxykynurenine, nmol/L	41.9 (35.2, 51.7)	46.2 (39.2, 53.4)	61.3 (49.7, 77.5)	77.7 (61.1, 102.3)	59.0 (46.2, 77.8)	47.9 (38.3, 55.1)	43.5 (36.3, 51.4)	105.0 (84.1, 132.0)
Kynurenic acid, nmol/L	46.1 (35.9, 60.4)	23.8 (21.2, 29.7)	23.0 (18.8, 27.6)	25.3 (19.5, 30.5)	40.2 (33.8, 50.6)	48.3 (40.8, 57.6)	48.8 (39.4, 61.2)	49.6 (40.4, 58.0)
Anthranilic acid, nmol/L	12.7 (10.5, 15.8)	11.1 (9.3, 12.8)	10.8 (8.8, 13.0)	11.6 (9.5, 13.8)	15.0 (13.7, 18.8)	16.3 (14.1, 19.5)	15.5 (12.9, 19.4)	22.2 (18.0, 28.1)
3-Hydroxyanthranilic acid, nmol/L	47.1 (38.0, 59.7)	60.3 (50.8, 71.1)	90.4 (77.4, 102.3)	109.5 (91.4, 127.0)	48.2 (39.5, 63.0)	40.3 (32.7, 52.9)	42.3 (36.0, 48.5)	42.2 (35.1, 53.1)
Xanthurenic acid, nmol/L	18.9 (13.8, 27.1)	22.0 (17.0, 27.4)	20.1 (14.3, 25.3)	18.6 (12.1, 25.5)	14.7 (10.7, 18.2)	16.5 (12.7, 22.4)	17.2 (13.6, 22.1)	7.2 (5.2, 9.7)

^1^HKr: 3-Hydroxykynurenine/(Kynurenic acid + Anthranilic acid + 3-Hydroxyanthranilic acid + Xanthurenic acid). The ratio was multiplied by 100

The kynurenines changed during pregnancy and postpartum and the concentrations differed from never-pregnant women at all-time points (*p* ≤ 0.001) (Table 2). In pregnancy week 36, median HAA was increased by 148% and HK by 93% compared to the controls, but both metabolites were reduced postpartum. Median KA was reduced in pregnancy (46% in week 18), but was higher than the controls at 4 and 6 months postpartum (*p* = 0.03). Compared to never-pregnant women, median maternal plasma AA was lower during pregnancy and higher postpartum, while the opposite pattern was seen for XA.

### Changes in the metabolic marker HKr in never-pregnant, pregnant and postpartum women

Maternal median HKr was significantly higher compared to the controls from pregnancy week 18 and increased further to 6 weeks postpartum (*p* ≤ 0.001) and thereafter normalized (Table 2).

Plasma PLP and HKr was negatively correlated in both never-pregnant (rho:− 0.51, *p* < 0.001), pregnant (rho:− 0.30 to − 0.41, *p* < 0.001) and postpartum women (rho:− 0.43, *p* < 0.001).

**Fig. 2:**
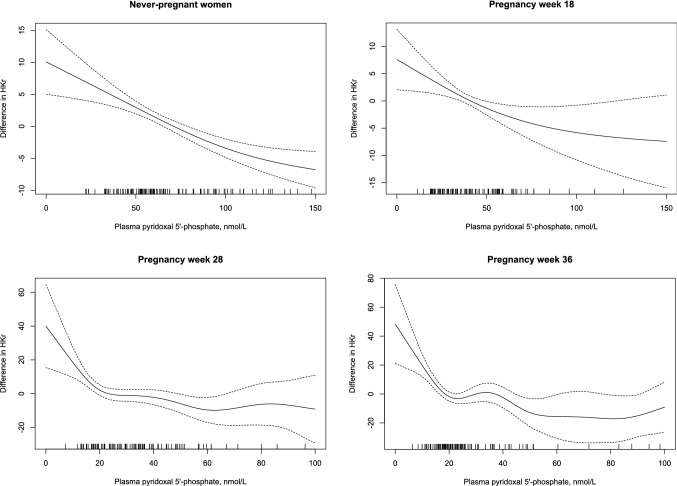

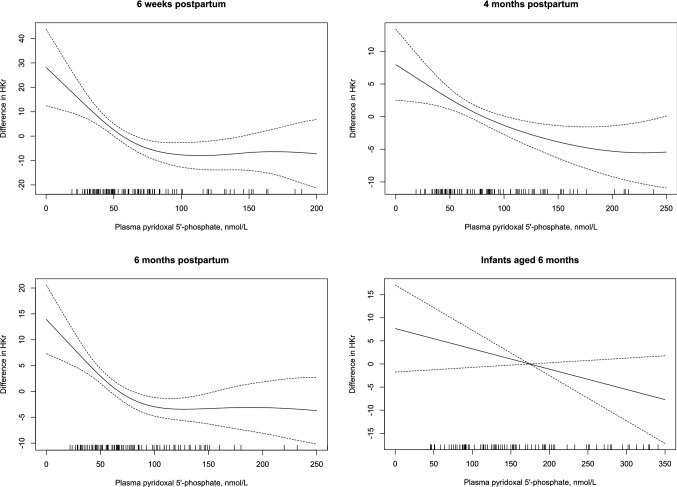
Plasma pyridoxal 5´-phosphate concentrations in relation to HKr (hydroxykynurenine/(kynurenic acid + anthranilic acid + xanthurenic acid + hydroxyanthranilic acid) in never-pregnant (*n* = 127), pregnant and postpartum women (*n* = 114) and their infants (*n* = 94) by generalized additive models (GAM). The values on the y-axes represents the difference from the respective mean HKr

In never-pregnant women, HKr started to increase when plasma PLP fell below 100 nmol/L (Fig. 2).

In pregnancy week 28 and 36, we observed a steep increase in HKr with plasma PLP concentrations below 30 nmol/L. Postpartum, a steep increase in HKr became evident when plasma PLP fell below 100 nmol/L (Fig. 2).

Based on this visual observation, we explored how low plasma PLP (< 30 nmol/L) in pregnancy week 28, was associated with changes in plasma PLP and HKr during pregnancy and postpartum. Table 3 shows that plasma PLP < 30 nmol/L in pregnancy week 28 was associated with lower plasma PLP and a higher HKr throughout pregnancy and postpartum.

**Table 3:** Maternal and infant plasma pyridoxal 5-phosphate and HKr in relation to maternal plasma pyridoxal 5-phosphate in pregnancy week 28

Plasma parameters Median (25th, 75th percentile)	Maternal plasma pyridoxal 5-phosphate in pregnancy week 28		*P* value^1^
	≤ 30 nmol/L *N* = 57	> 30 nmol/L *N* = 57	
Pregnancy week 18			
Pyridoxal 5-phosphate	29.9 (23.3, 39.0)	53.1 (40.2, 64.8)	< 0.001
HKr^2^	41 (33, 46)	34 (30, 43)	0.05
Pregnancy week 28			
Pyridoxal 5-phosphate	20.7 (16.3, 25.0)	41.6 (34.4, 51.2)	< 0.001
HKr^2^	44 (34, 53)	39 (30, 48)	0.03
Pregnancy week 36			
Pyridoxal 5-phosphate	18.0 (15.1, 23.0)	28.3 (22.5, 42.3)	< 0.001
HKr^2^	50 (43, 66)	41 (32, 51)	0.003
Postpartum 6 weeks			
Pyridoxal 5-phosphate	48.8 (38.6, 66.8)	80.2 (62.3, 119.8)	0.001
HKr^2^	51 (45, 64)	40 (33, 53)	0.001
Postpartum 4 months			
Pyridoxal 5-phosphate	58.1 (43.5, 90.3)	102.0 (70.0, 137.5)	< 0.001
HKr^2^	39 (33, 44)	34 (30, 40)	0.009
Postpartum 6 months			
Pyridoxal 5-phosphate	63.2 (46.1, 86.9)	107.5 (64.9, 146.8)	< 0.001
HKr^2^	36 (31, 42)	34 (28, 38)	0.05
Infants at 6 months			
Pyridoxal 5-phosphate	131.5 (91.3, 197.8)	179.5 (116.3, 260.3)	0.02
HKr^2^	90 (75, 111)	81 (56, 100)	0.03

^1^Comparison by Mann–Whitney *U* Test

^2^HKr: 3-Hydroxykynurenine/(Kynurenic acid + Anthranilic acid + 3-Hydroxyanthranilic acid + Xanthurenic acid). The ratio was multiplied by 100

### Factors related to plasma PLP and HKr in never-pregnant, pregnant and postpartum women

Regular users of MMN both during pregnancy and postpartum (*n* = 54) had significantly higher plasma PLP during the whole period compared to occasional users and non-users (*n* = 60) (*p* < 0.001) (supplemental Table 1). HKr was lower in regular users during pregnancy compared to occasional and non-users, but no significant differences were observed postpartum (*p* > 0.19) (supplemental Table 1). Never-pregnant women who were regular users of MMN had significantly higher median plasma PLP (103 (IQR 62, 133) versus 60 (49, 88) nmol/L, *p* = 0.002) and lower median HKr (30 (25, 35) versus 34 (29, 39), *p* = 0.02) compared to occasional and non-users of MMN supplements.

BMI was negatively correlated to maternal plasma PLP in pregnancy week 18 (rho:-0.23, *p* = 0.02), but not in never-pregnant women (rho: − 0.11, *p* = 0.19). No correlations were observed for BMI and HKr in either group (*p* > 0.5).

Mothers who were exclusively breastfeeding for a longer period had significantly higher HKr, whereas their plasma PLP concentrations did not significantly differ (Table 4).

**Table 4:** Infant and maternal plasma pyridoxal 5-phosphate and HKr at 6 months postpartum in relation to exclusive breastfeeding

Plasma parameters, median (25th, 75th percentile)	Months of exclusive breastfeeding			*P* value^1^
	0–1.9 months	2.0–4.9 months	5.0–6.0 months	
Infants, aged 6 months	*N* = 15	*N* = 67	*N* = 22	–
Pyridoxal 5-phosphate, nmol/L	215.0 (189.8, 313.5)	142.0 (96.5, 205.0)	111.0 (85.9, 173.5)	< 0.001
HKr^2^	76 (57, 108)	91 (74, 115)	81 (58, 95)	0.14
Mothers, 6 months postpartum	*N* = 16	*N* = 72	*N* = 25	–
Pyridoxal 5-phosphate, nmol/L	75.2 (63.7, 126,3)	78.3 (51.8, 117.0)	81.6 (47.6, 133.0)	0.94
HKr^2^	32 (28, 34)	34 (29, 40)	38 (34, 41)	0.008

^1^Comparison by Kruskal–Wallis test

^2^ HKr: 3-Hydroxykynurenine/(Kynurenic acid + Anthranilic acid + 3-Hydroxyanthranilic acid + Xanthurenic acid). The ratio was multiplied by 100

### Plasma PLP and kynurenines in infants at 6 months of age

Median infant plasma PLP and HKr were 2–2.5 fold higher compared to the never-pregnant women and the mothers at 6 months postpartum (Table 2). The higher HKr was mediated by higher 3-HK concentrations and lower XA concentrations in the infants compared to the women (Table 2).

There was only a weak negative, non-significant association between infant plasma PLP and HKr (rho:− 0.13, *p* = 0.21) (Fig. 2). Infant plasma PLP was invariably positively correlated to maternal PLP (rho: 0.10–0.28, *p* < 0.3) and negatively to maternal HKr (rho: − 0.28 to − 0.07, *p* < 0.5) both during pregnancy and postpartum. Infant plasma PLP was lower and HKr higher if maternal plasma PLP was < 30 nmol/L in pregnancy week 28 (Table 3).

**Fig. 3:**
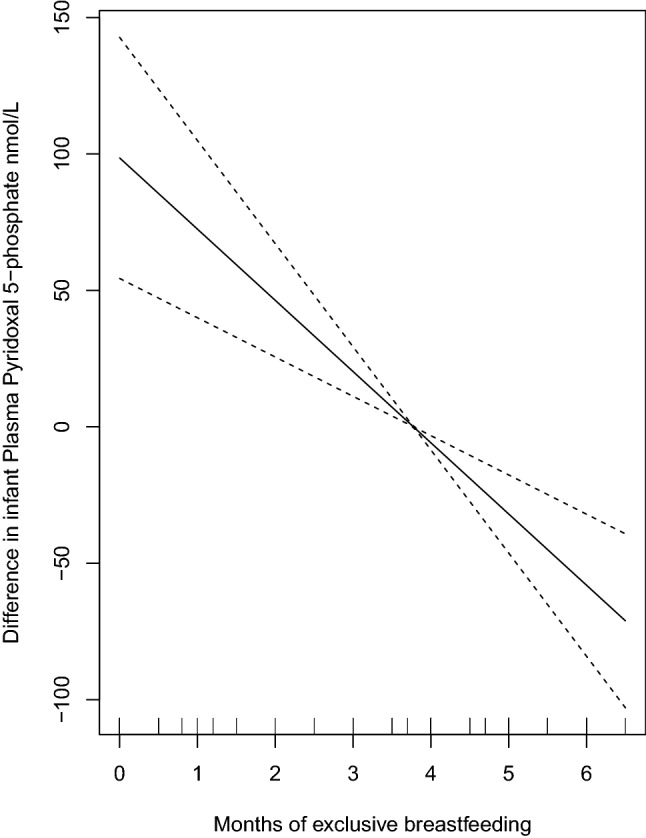
Plasma pyridoxal 5´-phosphate concentrations in relation to months of exclusive breastfeeding in infants aged 6 months (*n* = 104) corrected for maternal plasma PLP in pregnancy week 28 and use of MMN throughout pregnancy and lactation, by generalized additive models (GAM). The values on the y-axes represents the difference from the respective mean plasma pyridoxal 5´-phosphate concentration

Median infant plasma PLP was lower with increasing number of months of exclusive breastfeeding (Table 4), even when corrected for maternal plasma PLP in pregnancy week 28 and use of MMN throughout pregnancy and lactation (Fig. 3).

The strongest predictor of infant plasma PLP was months of exclusive breastfeeding followed by maternal plasma PLP in pregnancy week 28, in a multiple linear regression model, which additionally included gender, birth weight, percentage weight increase to 6 months and maternal age, use of MMN during pregnancy and postpartum and parity. No significant predictors were seen for infant HKr in the same model (Table 5).

**Table 5:** Determinants of infant plasma pyridoxal 5-phosphate and HKr concentrations by multiple linear regression

Variables included in the model	Plasma pyridoxal 5-phosphate, nmol/L			HKr^1^		
	Beta	B	*P* value	Beta	B	*P* value
Months of exclusive breastfeeding^2^	− 0.321	− 56.6	0.001	− 0.038	− 1.8	0.69
Birth weight	− 0.137	− 0.03	0.24	0.030	0.002	0.82
Percentage weight increase to 6 months	0.073	0.26	0.55	− 0.016	− 0.016	0.95
Maternal plasma pyridoxal 5-phosphate in pregnancy week 28	0.225	1.16	0.04	− 0.236	− 0.34	0.06
Maternal use of multiple micronutrient supplements^3^	0.092	13.36	0.39	− 0.048	− 1.98	0.69

The model additional included gender, maternal age and parity

^1^HKr: 3-Hydroxykynurenine / (Kynurenic acid + Anthranilic acid + 3-Hydroxyanthranilic acid + Xanthurenic acid). The ratio was multiplied by 100

^2^Months of exclusive breastfeeding categorized; 0–1.9 months (*n* = 15), 2–4.9 months (*n* = 67), 5–6 months (*n* = 22)

^3^Maternal use of multiple micronutrient supplements during pregnancy and postpartum was categorized as; Never user (*n* = 15), Occasional user (*n* = 45), Regular user (*n* = 54)

## Discussion

Compared to never-pregnant women, plasma PLP decreased during pregnancy and increased postpartum, whereas HKr was increased from pregnancy week 18 to 6 weeks postpartum, indicating functional vitamin B6 insufficiency in the mother during this period. In never-pregnant women and in pregnancy week 18, HKr increased when plasma PLP fell below 100 nmol/L.

A plasma PLP < 30 nmol/L in pregnancy week 28 was associated with significantly lower plasma PLP and higher HKr in the mothers throughout pregnancy and postpartum and in the infants at 6 months.

Infants aged 6 months had the highest concentrations of both plasma PLP and HKr. Exclusive breastfeeding for more than 2 months and maternal plasma PLP < 30 nmol/L in pregnancy week 28 was associated with lower infant plasma PLP concentrations.

### Metabolically adequate plasma PLP concentrations in never-pregnant, pregnant and lactating women

A plasma PLP concentration above 30 nmol/L is considered to be an adequate vitamin B6 status in adults [1]. However, the metabolic marker HKr is reported to increase already when plasma PLP falls below 100–120 nmol/L, with a steeper increase below 50 nmol/L [10, 15]. We observed a similar pattern in never-pregnant women, indicating that adults need a plasma PLP concentration in the range of 50–100 nmol/L to have a metabolically adequate vitamin B6 status.

Plasma PLP is reported to decrease throughout pregnancy [5], and in our population median plasma PLP was reduced by 39% already in pregnancy week 18 and by 65% in week 36, compared to never-pregnant women. During pregnancy, maternal blood volume increases by up to 40–50%, starting from week 6–8, reaching a peak in week 32 [16], and causing hemodilution resulting in lower blood levels of several biochemical analytes [17]. Whether the observed reduction is due to pregnancy-induced physiological responses or indicate functional intracellular PLP depletion, due to placental transfer to the fetus, is unknown.

In pregnancy week 28, HKr increased steeply when plasma PLP fell below 30 nmol/L, and a plasma PLP concentrations above this level in week 28 was associated with a higher vitamin B6 status throughout pregnancy and postpartum. Thus, a plasma PLP concentration > 30 nmol/L in mid-pregnancy might be required to secure a metabolically adequate vitamin B6 status during pregnancy and lactation.

Postpartum the plasma PLP concentrations increased substantially, in both regular, occasional and non-users of MMN. The observed increase in plasma PLP postpartum resemble the observed postpartum increase in serum cobalamin, and may be a physiological process for increasing the vitamin concentrations in the breast milk [18].

There are currently no previous data on the functional marker HKr during pregnancy and lactation. We show that plasma PLP and HKr correlate both in pregnant and postpartum women, indicating that HKr reflects vitamin B6 status also during these life periods. Median HKr increased during pregnancy and was significantly higher in the mothers from pregnancy week 18 to 6 weeks postpartum, compared to never-pregnant controls, indicating maternal non-optimal vitamin B6 status. HKr was lower in regular users of MMN compared to occasional and non-users of MMN during pregnancy, but not postpartum. Postpartum, both users and non-users of MMN supplements had very high plasma PLP concentrations. An increase in HKr became evident when plasma PLP fell below 100 nmol/L possibly indicating maternal non-optimal intracellular PLP status. Kynurenine metabolism is complex and changes in hormones, like estrogen concentrations, which appear both during pregnancy and postpartum [19, 20], will have an impact on kynurenine metabolites and HKr and complicate the relation to PLP concentrations in both pregnant and postpartum women.

The maternal plasma PLP concentrations in pregnancy week 18 in our population are somewhat higher than in a study from the Norwegian MoBa cohort. The MoBa cohort had a median plasma PLP of 29.3 (IQR 21.9–42.0) if they were folate supplement users, of which 40% reported taking additional vitamin B6 supplements [21], compared to 24.1 (18.6–30.4) nmol/L in non-users [22]. Non optimal blood sample handling is known to cause a reduction in PLP concentration [23], and as the blood samples in the MoBa study were collected throughout Norway and sent by regular mail [24], the plasma PLP concentrations in the MoBa study may be falsely low.

A varied omnivore diet is reported to provide a daily amount of 6–9 mg vitamin B6, considered to be adequate for adults according to one study [25]. However, reported intakes vary considerably in the literature, mainly due to the variable use of supplements but also to the dietary assessment methods used. Average total vitamin B6 intake ranged from 1.4 to 3.1 mg/day in adults from nine countries in Europe [26]. The estimated mean intake of vitamin B6 based on self-reported questionnaires from more than 17,000 pregnant women in the MoBa study was 4.6 (SD 11.0) mg of which 3.3 (SD 11.0) mg came from supplements.

Recent recommendations from the Nutrition Societies of Germany, Austria, and Switzerland set an Average Requirement (AR) for vitamin B6 of 1.2 mg/day for adult females, 1.3 mg/day for pregnant women in the first trimester and 1.5 mg/day in the second and third trimesters, and 1.3 mg/day for lactating women, to ensure a plasma PLP concentration of ≥ 30 nmol/L [27].

In our population, we did not have data on estimated intake of vitamin B6 from the diet, but the most commonly used MMN contained only 1.4 mg vitamin B6 during pregnancy and 1.5 mg postpartum, and as we show this had a significant effect on plasma PLP concentrations. However, even in regular MMN users, HKr increased during pregnancy to 6 weeks postpartum, indicating insufficient vitamin B6 to support optimal kynurenine metabolism.

The effect of daily vitamin B6 supplement doses was evaluated in a group of pregnant women with a mean dietary vitamin B6 intake of 1.43 ± 1.28 mg/day [28], which was equivalent to the dietary intake in the MoBa study. Cord plasma PLP levels reached a maximum when maternal supplementation was ≥ 7.5 mg and the same dose was also required to prevent a decrease in maternal plasma PLP at delivery. The authors concluded that a vitamin B-6 intake between 5.5 and 7.6 mg/day (diet plus supplement as pyridoxine equivalents) is required to maintain maternal plasma PLP levels at term at a level comparable to the values at the first visit (mean pregnancy week 15 ± 4 weeks) [28].

### Determinants of vitamin B6 status in infants

Plasma PLP is reported to be substantially higher in newborns compared to their mothers [5], which is in agreement with our results. In a study published in 1992, the maternal plasma PLP concentration was 22.2 nmol/L, whereas in venous cord plasma the concentration was 112.1 nmol/L [7], suggesting fetal sequestration of the vitamin. In the present study, infants had the highest concentrations of both plasma PLP and HKr. The weak correlation between plasma PLP and the metabolic marker in newborns could be due to age-dependent changes in the kynurenine metabolic pathway or related to the fact that the metabolic marker does not change substantially when plasma PLP is > 100 nmol/L.

In children, plasma PLP is reported to progressively decrease throughout the first year and then more slowly throughout life [29]. Infant diet during the first months of life seems to modify this pattern. A significant positive association has been reported between the age of the infant in months and total vitamin B6 concentration in breast milk [30]. Vitamin B6 intake of breastfeeding mothers has been shown to be a strong predictor of infant B6 status [9]. We did observe positive correlations between infant and maternal plasma PLP from 6 weeks to 6 months postpartum, however, the strongest predictor of infant plasma PLP was months of exclusive breastfeeding, followed by maternal plasma PLP in pregnancy week 28. Despite correcting for maternal vitamin B6 status and use of MMN, infant plasma PLP decreased with months of exclusive breastfeeding. However, median plasma PLP was above 100 nmol/L independent of breastfeeding period and the metabolic marker did not differ according to months of breastfeeding. This indicates that most infants had an adequate PLP status, though lower in infants who were longer breastfed.

In formula fed infants with a birth weight from 2.5 to 3 kg, median plasma PLP decreased from 274 (IQR 201, 337) nmol/L at 6 weeks to 184 (123, 278) nmol/L at 6 months. In the exclusively breast-fed infants, median plasma PLP increased from median 79 (42, 132) nmol/L at 6 weeks to median 122 (92, 162) nmol/L at 6 months of age [31]. At all-time points, the breast-fed infants had significantly lower plasma PLP concentrations (*p* = 0.001) [31]. In our study, plasma PLP were almost 50% lower, but still > 100 nmol/L, in infants who were exclusively breastfed for 5 to 6 months, compared to those who were breastfed less than 2 months, but the concentrations were still far above the assumed adequate level in adults (30 nmol/L).

In breast-fed Finnish infants, vitamin B6 status was generally adequate independently of the actual vitamin status of the nursing mother during the first 4 months. Most of the infants with low status at 2 months were born to mothers who were not using MMN supplements during pregnancy. After 6 months of exclusive breastfeeding, 30% of the infants had low vitamin B6 status, and thereafter the risk of low vitamin B6 status increased even if the mother's status was adequate [32].

### The importance of vitamin B6 status in infants

It is unclear whether a lower vitamin B6 status may affect infant neurodevelopment. In animal studies, severe maternal vitamin B6 deficiency has been associated with lower body weight, skeletal defects, reduced neuronal connections, decreased myelination, convulsions and impaired neuromotor development in the offspring [3, 4, 33], but no such associations have been documented in humans [5]. A systematic review published in 2021 reported only on small, cross-sectional studies on the association between vitamin B6 in breast milk and neurodevelopment in neonates [34]. In one study, infant scores on habituation and autonomic stability were positively correlated with milk pyridoxal values at 8–11 days postpartum (*n* = 25) [35]. In Egyptian infants, consolability and response to aversive stimuli at age 3 to 6 months were significantly correlated with maternal vitamin B6 status, and mothers with marginal B6 status were found to be less responsive to their infants' vocalizations and showed less effective intervention to infant distress [36].

### Strength and limitations

This study was designed with longitudinal measurements during pregnancy and postpartum period, including infants at 6 months, with no loss to follow- up. Clinical data were collected by questionnaires, prone to recall bias, but the same two doctors did all the interviews throughout the study period. Neither maternal nor infant dietary intake of B6 from food or breastmilk were assessed, which is a limitation to the study.

Due to technical issues, blood samples were missing from 11 of the 114 infants at 6 months, but there were no differences in maternal plasma PLP and HKr concentrations during pregnancy and postpartum between these mothers and the mothers of the 103 infants who gave a blood sample (*P* > 0.2).

## Conclusion

An adequate vitamin B6 status is important for all age-groups. Based on observed changes in the metabolic marker HKr, a plasma PLP > 50–100 nmol/L may secure an adequate vitamin B6 status in non-pregnant women, while a plasma PLP > 30 nmol/L in pregnancy week 28 may secure an adequate status during pregnancy and postpartum. Regular maternal use of vitamin B6 supplements during pregnancy and postpartum is associated with higher maternal plasma PLP and lower HKr, while months of exclusive breastfeeding is the most important factor for reduced infant plasma PLP concentrations.

## Supplementary Information

Below is the link to the electronic supplementary material.

Supplementary file1 (DOCX 15 KB)

## Abbreviations

AA: Anthranilic acid
BMI: Body mass index
GAM: Generalized additive models
HAA: Hydroxyanthranilic acid
HKr: 3-Hydroxykynurenine/(kynurenic acid + anthranilic acid + xanthurenic acid + hydroxyanthranilic acid)
KA: Kynurenic acid
MMN: Multiple micronutrients
PLP: Pyridoxal 5´-phosphate
SD: Standard deviation
XA: Xanthurenic acid
