# Supplementary material for: Assessment of vitamin B6 status in never-pregnant, pregnant and postpartum women and their infants
Source: Eur J Nutr. 2022 Nov 1;62(2):867–78. doi: 10.1007/s00394-022-03033-4 (PMC9941241; doi:10.1007/s00394-022-03033-4)
Supplement: Supplementary file 1 — Supplementary file1 (DOCX 15 KB) [file 394_2022_3033_MOESM1_ESM.docx]

| **Supplemental Table 1. Plasma pyridoxal 5-phosphate and HKr concentrations in pregnant and postpartum women according to use of multiple micronutrient supplements during pregnancy and postpartum** | | | | | | | | | | | | |
| --- | --- | --- | --- | --- | --- | --- | --- | --- | --- | --- | --- | --- |
| Parameters  Median  (25th, 75th percentile) | Pregnancy | | | | | | Postpartum | | | | | |
|  | Week 18 | | Week 28 | | Week 36 | | 6 weeks | | 4 months | | 6 months | |
|  | MMN+/-^1^ | MMN++^2^ | MMN+/-^1^ | MMN++^2^ | MMN+/-^1^ | MMN++^2^ | MMN+/-^1^ | MMN++^2^ | MMN+/-^1^ | MMN++^2^ | MMN+/-^1^ | MMN++^2^ |
| Pyridoxal 5-phosphate, nmol/L | 32.5  (23.4, 45.5) | 51.0  (33.3, 65.8) | 23.7  (17.3, 32.0) | 38.0  (29.0, 51.1) | 18.5  (15.5, 23.6) | 27.5  (22.1, 39.3) | 49.3  (38.3, 72.4) | 82.0  (54.3, 124.5) | 58.9  (45.7, 89,0) | 104.0  (83.5, 137.0) | 64.8  (45.6, 89,2) | 109.0  (64.0, 147.5) |
| P value | <0.001 | | <0.001 | | <0.001 | | <0.001 | | <0.001 | | <0.001 | |
| HKr^3^ (no unit) ^1^ | 40  (33, 47) | 34  (31, 43) | 47  (35, 56) | 38  (31, 44) | 51  (40, 71) | 42  (32, 51) | 47  (41, 58) | 45  (36, 59) | 38  (33, 43) | 34  (31, 42) | 36  (30, 41) | 34  (30, 38) |
| P value | 0.08 | | 0.006 | | 0.004 | | 0.44 | | 0.19 | | 0.27 | |

^1^MMN +/-: Occasional user of multiple micronutrient supplements (n=45) and non-user (n=15)

^2^MMN++: Regular user of multiple micronutrient supplements (n=54),

^3^HKr: 3-Hydroxykynurenine / (Kynurenic acid + Anthranilic acid + 3-Hydroxyanthranilic acid + Xanthurenic acid). The ratio was multiplied by 100.
